# Plant-Based Alternatives to Yogurt: State-of-the-Art and Perspectives of New Biotechnological Challenges

**DOI:** 10.3390/foods10020316

**Published:** 2021-02-03

**Authors:** Marco Montemurro, Erica Pontonio, Rossana Coda, Carlo Giuseppe Rizzello

**Affiliations:** 1 Department of Soil, Plant, and Food Science, University of Bari Aldo Moro, 70126 Bari, Italy; marco.montemurro@uniba.it (M.M.); erica.pontonio@uniba.it (E.P.); 2 Department of Food and Nutrition, University of Helsinki, 00014 Helsinki, Finland; rossana.coda@helsinki.fi; 3 Helsinki Institute of Sustainability Science, 00014 Helsinki, Finland; 4 Department of Environmental Biology, “Sapienza” University of Rome, 00185 Rome, Italy

**Keywords:** milk alternatives, lactic acid bacteria, yogurt-like, plant-based foods

## Abstract

Due to the increasing demand for milk alternatives, related to both health and ethical needs, plant-based yogurt-like products have been widely explored in recent years. With the main goal to obtain snacks similar to the conventional yogurt in terms of textural and sensory properties and ability to host viable lactic acid bacteria for a long-time storage, several plant-derived ingredients (e.g., cereals, pseudocereals, legumes, and fruits) as well as technological solutions (e.g., enzymatic and thermal treatments) have been investigated. The central role of fermentation in yogurt-like production led to specific selections of lactic acid bacteria strains to be used as starters to guarantee optimal textural (e.g., through the synthesis of exo-polysaccharydes), nutritional (high protein digestibility and low content of anti-nutritional compounds), and functional (synthesis of bioactive compounds) features of the products. This review provides an overview of the novel insights on fermented yogurt-like products. The state-of-the-art on the use of unconventional ingredients, traditional and innovative biotechnological processes, and the effects of fermentation on the textural, nutritional, functional, and sensory features, and the shelf life are described. The supplementation of prebiotics and probiotics and the related health effects are also reviewed.

## 1. Introduction

In recent years, novel plant-based (PB) foods and beverages have been designed and made available for the market to satisfy the increasing demand for alternatives to the animal-derived products. Milk and dairy products have been considered for a long time as a class of food with essential compounds for human nutrition, which are hardly found, with the same balance, in others. However, people suffering by health problems related to high cholesterol intake in diet, lactose intolerance, or malabsorption, and allergy to milk proteins, should consume alternative products. Moreover, the overall consumers’ awareness about the effects of their food choices on environment and health, and the growing trend of vegetarianism, in addition to the limited use of dairy products in some areas, are leading to higher demand for PB products.

Plant-derived protein consumption is continuously increasing in Europe with a yearly growth of the 11% of the plant foods and beverages alternative to dairy products [1]. Despite the production of non-dairy beverages has a long tradition [2,3], the design of novel PB and yogurt-like (YL) products (PBYL) gained high interest due to the new opportunities offered by the worldwide market [4]. Furthermore, PBYL are considered an economic alternative to dairy products in developing countries [2].

With PBYL, we refer to vegetable products similar to the conventional yogurt in terms of textural and sensory properties and ability to host viable lactic acid bacteria for a long-time storage. Fermentation applied to PB matrices has been identified as a natural and effective biotechnological option to increase their technological, sensory, nutritional, and functional properties [5,6], thus meeting both consumers and food industry demand.

Conventional yogurt is made through the fermentation of cow milk by *Streptococcus thermophilus* and *Lactobacillus delbrueckii* subsp. *bulgaricus* until pH lower than 4.5 and final lactic acid bacteria (LAB) density higher than 8 log10 cfu/g are reached [7]. Through acidification, fermentation directly impacts the stability of casein micelles, reducing their charge, dissolving some of the insoluble calcium phosphate crosslinks, and modifying internal bonding between proteins. The achievement of a pH value lower than the isoelectric point of caseins causes their gelation. The formation of this cohesive protein network represents one of the main issues characterizing the production of YL products with alternative vegetable ingredients, these latter characterized by proteins of different nature, not easily precipitable by acidification [3,8]. PBYL are generally made by fermenting aqueous extracts or flour–water suspensions of cereal, pseudocereals, legumes, and nut flours, or homogenized fruit pulps [9]. Several attempts to obtain a yogurt-comparable protein structure were performed in recent years on PBYL. However, (1) the low amount of proteins, (2) the different coagulation properties, and (3) the need for the addition of structuring agents and emulsifiers, often make processes expensive and time consuming. Moreover, the destabilization of the plant protein structure caused by fermentation and acidification, can lead to the weakening of the product structure and to aqueous phase separation during storage [10]. Therefore, the optimal texture achieved in commercial non-dairy products is usually obtained with additives (protein extracts, inulin, thickeners, and emulsifier) which do not meet the growing trend of clean label products [3].

Aiming at formulating products without additives inclusion, the use of exopolysaccharide (EPS)-producing bacteria as starters for PBYL fermentation is one of the most investigated alternatives. The in-situ EPS production leads to the improvement of textural, sensorial, nutritional, and functional properties of PBYL [11,12].

Starch gelatinization via the application of proper heating treatments was proposed to increase the viscosity of plant ingredients before fermentation, thus also preventing phase separation and decreasing the entity of endogenous microbes before starter inoculation [13,14].

The nutritional value of PBYL products is mainly due to the raw materials included in the formulations. Cereals (e.g., oat, rice, maize, wheat, barley) are largely employed as main ingredients of the PBYL recipes, due to the global availability and the moderate cost, identifying them as the main source of macro- and micro-nutrients worldwide. Pseudocereals and legumes are protein sources alternative to animal-derived ingredients, characterized by abundance of proteins with high biological value, fibers and bioactive compounds (see [15] for review); thus, they were largely investigated as novel PBYL ingredients. However, the nutritional and functional value of these matrices could be lowered because of the presence of antinutritional factors (ANF) which could also negatively affect the sensory profile of the products. Common plants ANF are condensed tannins, saponins, phytic acid, α-galactosides, and trypsin inhibitors [16]. Fermentation has been widely explored as a bioprocess able to reduce the ANF impact, besides positively affecting nutritional, sensory, and technological properties of plant-derived ingredients [15]. A proper selection of microbial starters was recognized of primary importance to obtain high quality products. LAB, thanks to their metabolic adaptability and the safe and traditional use in food fermentations, are considered the best candidates for this role [17]. Compared to yeasts, LAB do not produce or produce low ethanol, thus are suitable starters for functional foods and beverages making, in which alcohol is not allowed [18]. Furthermore, lactic acid fermentation confers to the matrix the acidic sensory profile characterizing conventional milk-based yogurt.

LAB activity is not only related to acidification, since several enzymatic activities lead to an efficient proteolysis, increased contents of different bioactive compounds, and the decrease of ANF [19], besides sensory quality improvement. Moreover, considering the debate on the poor survival in the digestive tract of the starters used in conventional yogurt making (*Str. thermophilus* and *Lb. delbrueckii* spp. *bulgaricus*) [20], specifically formulated PBYL products have also been proposed as carriers for selected probiotic strains [21].

In this article, the production options and the textural, nutritional, functional, and sensory properties of fermented PBYL products, without any addition of dairy constituents (e.g., lactose, whey proteins, caseinates), are reviewed. Compared to conventional yogurt, drinkable yogurt-like products, representing a beverage category with several differences in technological and nutritional characteristics, and consumption habits, are not included in this investigation. The updated nomenclature of the species belonging to the genus *Lactobacillus* [22] has been used in the text.

## 2. Ingredients

The selection of suitable ingredients is the first step to set up the formulation of a PBYL. Typically, cereals, pseudocereals, and legumes are used in different combination to achieve optimal textural and nutritional quality of products (Table 1).

Cereals are widely used in traditional formulation of PB beverages in Africa and Asia (such as *boza*, *bushera*, *chhang*, *chica*, *haria*, *mahewu*, *omegisool*, *pozol*, *togwa*), and represent the principal ingredients also for innovative production of PBYL [2,46]. Cereals are a good source of nutrients including vitamins, minerals and fibers [47], however, the increasing prevalence of celiac sprue and other diseases related to gluten assumption led towards the investigation of gluten free alternative flours [48].

Among cereals, oat is largely employed for making experimental and commercial YL products [2,34,49,50]. It is a good source of unsaturated fatty acids, high quality proteins and natural antioxidants (e.g., tocols, phenolic compounds, and avenanthramides) [34]. Moreover, the positive effects of oat fiber, thanks to the presence of β-glucans, were correlated with the reduction of the blood glucose rise after meal and the reduction of blood cholesterol [51]. Although still debated, oat could be included in the gluten free diet [52]. Due to all these benefits, oat has been widely used for making PBYL with functional properties [53,54]. However, due to the unpleasant taste (mainly related to lipid oxidation derivatives) thermal treatment is needed to increase sensory acceptability and to inactivate lipolytic enzymes [55].

Maize has also been proposed as main ingredient for making YL products [56], being one of the most important crop worldwide [57]. Moreover, maize is preferred to other cereals, from a nutritional point of view, thanks to the higher fat, iron and fiber content, if compared with wheat and rice. However, its protein quality is usually lower because of low lysine and tryptophan concentrations, which were increased by selecting maize cultivars described as quality protein maize (QPM) [57].

Rice is the most important crop in Asia, where it represents the most economic source of energy and protein. It is largely employed as YL ingredient thanks to its neutral taste and to the good aptitude to form a viscous gel after thermal treatment. However, it is mostly consumed as white rice while brown rice is nutritionally more complete providing also functional compounds (e.g., γ-oryzanol, γ-aminoburyric acid (GABA), and ferulic acid) which are mainly contained in germ and bran fractions [58].

Millet represents a low-cost staple food for a large portion of Asian and African population, where this crop is diffused thanks to the resistance to difficult growing conditions (e.g., water scarcity, disease, pests, and poor soils) [59]. Millet has been described as a promising YL ingredient, and it has been used in combination with other PB materials and structuring agents [60].

Pseudocereals, staple food in several parts of the world, are cultivated in different regions, thanks to the presence of a number of varieties and the adaptation to different environmental conditions. Among them, quinoa is one of the most widespread, characterized by high quality protein, optimal ratio of essential fatty acids and the presence of several functional compounds (for a review, see [61]). Several biotechnological processes for making quinoa-based YL were recently described in literature [33,35,62]

Among legumes, soybean has been widely used for making YL products, considering its high protein content and quality, its functional properties [63], and the good attitude to be fermented. Kellogg was the first food company to produce and market a soy PBYL fermented by *Lactobacillus acidophilus* [64]. Overall, commercial soymilk, is a good substrate for the growth of the yogurt starters, *Lb. delbrueckii* and *Str. thermophilus* [65]. Soy is nowadays the most used plant protein source in food industry because of its nutritional profile and low production costs [26]. Nevertheless, the beany flavor of soy-based products and the presence of allergens are still considered critical commercial issues. Additional problems related to large-scale soy employment recently emerged, such as the sustainability of the production chain, the diffusion of transgenic cultivars, and the import/export governance among producing (such as the USA) and importer countries (such as those in the European community) [66], thus pushing the global research to investigate other alternatives.

Considering the need to obtain a protein network similar to dairy yogurt, the technological potential of different plant-protein isolates as structuring agents in PBYL was investigated by several authors. Overall, plant protein isolates are considered texture improvers, thanks to the high solubility in water, the emulsifying activity, and the foaming property. Among cereal-derived ingredients, the use of oat protein concentrate (OPC) and oat protein isolate (OPI), was proposed [23]. Protein-rich fractions from legumes were also used in YL formulation. Lupin protein isolate was used as the main protein source in a formulation including glucose and coconut oil [11] while recently, a pea protein isolate was used to prepare a PBYL, showing its ability to form gels after heating and fermentation [25]. Potato protein isolate was shown to be suitable to fortify a PBYL [24]. Despite the low amount of protein in potato (2%) the isolate showed good nutritional value and technological properties [67].

## 3. Texture: Role of the Bioprocessing Options

### 3.1. Physical Treatments and Bioprocessing

Yogurt is typically a product characterized by creamy structure due to the proteins network obtained thanks to LAB fermentation of milk. In PBYL products, the achievement of similar textural properties—intended as proper viscosity, adherence to spoon, and organoleptic perception—represents the main technological challenge. Despite the addition of structuring agents like gums and hydrocolloids provide often reliable results, the scientific community and the food industry are evaluating more sustainable solutions potentially more acceptable by the modern consumer, that requires reduced presence of additives and clean-label foods.

**Figure 1:**
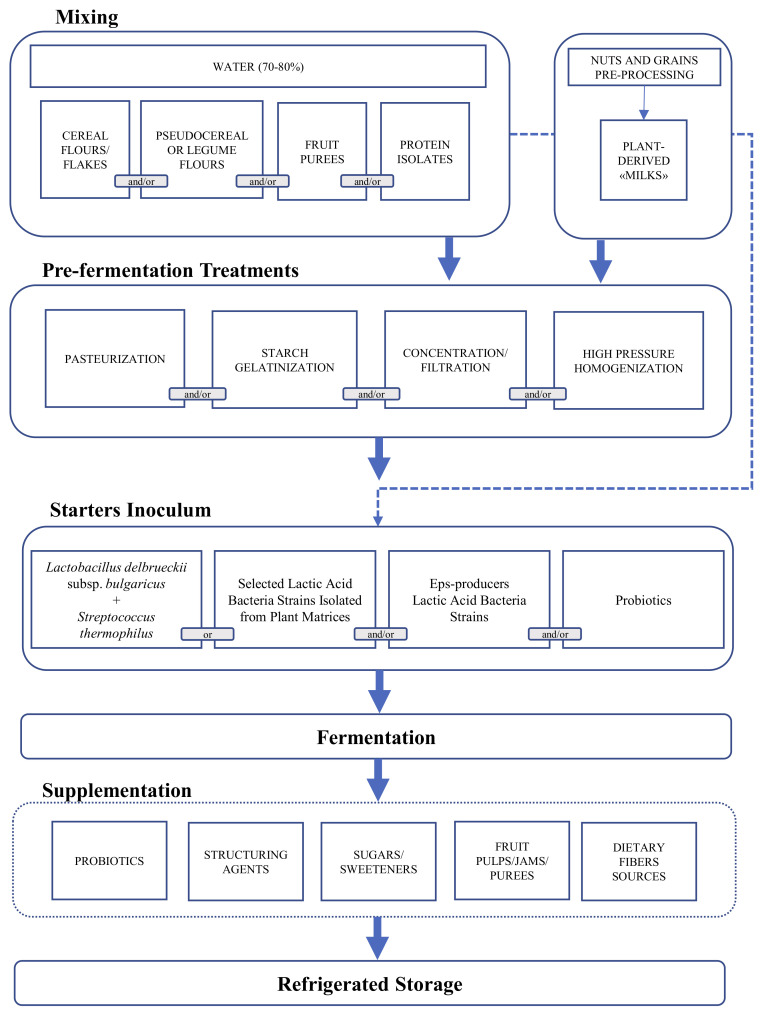
Flow chart of the plant-based yogurt-like production including the biotechnological options proposed by the recent scientific literature.

Therefore, several technological options—mainly based on the application of heat treatments, enzymes, and fermentation—were studied. Considering that raw materials could lack in several structuring compounds (e.g., low concentration or poor technological quality of proteins), the addition of PB protein isolates or concentrates in formulation was largely investigated. Overall, the setting up of PBYL formulations usually includes a combination of previously mentioned solutions schematized in (Figure 1).

The physicochemical and sensory properties of commercially available PBYL made from soy, coconut, cashew, almond, and hemp have been compared to a benchmark dairy yogurt [41]. Overall, the water holding capacity (WHC) and apparent viscosity of the dairy yogurt were lower than those of the PBYL (75.7% ± 0.68% and 0.24 Pa·s in yogurt vs. 82.8% ± 0.92%−99.3% ± 0.50% and 0.29–0.75 Pa·s, in PBYL). No correlation between protein contents of PBYL (ranging from 0.60 to 4.60 g/100g) and textural properties of products was observed while the presence of agar or hydrocolloids in formulation significantly affected their rheology. All the optimized PBYL formulations were characterized by higher values of all the parameters considered compared to dairy yogurt, confirming that due to their gelling properties, hydrocolloids, whether naturally contained in raw ingredients or added as additives, can substitute the protein network of yogurt [68].

The total solids amount of PB ‘milk’, intended as the concentration of flour and other ingredients to be converted into the final product, is an important parameter to obtain optimal texture of PBYL products. The Food and Drug Administration (FDA) set the limit of milk solids, which must be present in fermented yogurt corresponding to minimum 3.25% of fat and 8.5% of no-fat solids [69]. To obtain similar solids amount in PBYL products, concentration at high temperature was applied [26]. Heating soymilk at 90 °C allowed to reach proper solid percentage (11%) and the inactivation of undesired microorganisms. Heating together with fermentation were proven to be essential to obtain optimal soymilk yogurt texture. Heat treatments are usually applied to PB ingredients, in presence of water, to induce starch gelatinization and obtain the formation of a consistent gel texture. The setting up of temperature and time of the gelatinization process is strictly dependent on raw materials used and their composition. Heat-induced gelatinization causes the irreversible swelling of starch, leading to a significant increase of viscosity. However, this process is influenced by several variables that differ in each matrix, including the starch content, its amylose/amylopectin ratio, the molecule branching, and the granule structure (for a review, see [70]). Overall, starchy flours suspensions (e.g., emmer, oat, quinoa, rice, etc.) were used at different percentages in water, usually from 15% to 35%. This amount was selected to achieve optimal PBYL texture after gelatinization at high temperature [14,23,27,32,33,34]. Similar ratios were also used for cereal-legume blends including, for example, rice, soy, oat, barley and emmer flours [13], or rice, lentil, and chickpea flours [14].

Besides the application of heat-induced starch gelatinization, the use of combined technologies was reported, for example homogenization and thermal treatments. High-pressure homogenization (172 MPa for few seconds) coupled with thermal treatment at 85 °C for 30 min, led to the best texture of an almond PBYL [31]. The almond YL structure resulted stable for 28 days of storage at 4 °C [31]. It was reported that homogenization leads to the reduction of droplet size partially increasing protein solubilization [71] while the heat treatment was responsible for the formation of a denatured protein network involving lipids [72]. It was also observed that fermentation improved the cohesiveness of the starch gel, as the consequence of pH-induced denaturation of the proteins entrapped into the gelatinized matrix [23].

Ultra-high-pressure homogenization (UHPH), was used to obtain PBYL from potato protein isolate [24]. UHPH caused a relevant temperature increase (estimated in 14–25 °C per 100 MPa) resulting in a combination of homogenization and thermal treatment. The application of high-pressure treatments (>200 MPa) before the inoculum favored the rapid growth of the microbial starters in the matrix, probably as consequence of the higher availability of nutrients. UHPH treatment decreased the aqueous phase separation during storage of the product, in which sunflower oil (3%) was also added [24]. This result was in accordance with the hypothesis of increased hydrophobic interactions between proteins and fat globules after treatment, confirming the creation of an oil–water stable interface [73].

Ultra-high-temperature (UHT) treatment at 140 °C for 10 s or a pasteurization at 80 °C for 60 s, were applied to a lupin PBYL product containing 2% of lupin protein isolate, 4% of glucose and 4% of coconut oil [11]. PBYL were fermented by four different selected EPS-producing LAB (*Lacticplantibacillus plantarum* TMW 1.460 and TMW 1.1468, *Pediococcus pentosaceus* BGT B34, and *Levilactobacillus brevis* BGT L150). Apparent viscosity, WHC, and the hysteresis loop area resulted higher in PBYL produced through UHT treatment. Hysteresis loop area can be considered an index related to the gel capacity to regenerate after shear-induced structure breakdown. UHT-treated PBYL also resulted a better substrate for EPS production, since increases (from 23% to 53%) of the EPS concentration were found, compared to the corresponding pasteurized matrices. Textural analysis also showed increased firmness (intended as the maximum force needed for gel breaking) and consistency, when UHT treatment was used [74].

The importance of the concurrence of physical treatment and bioprocessing on the optimal textural properties of the PBYL products was reported in several studies. Lorusso and colleagues set-up a protocol for making a quinoa-based product by applying sequentially a thermal treatment to ensure starch gelation (at 63° C), followed by fermentation with EPS-producing LAB strains, thus obtaining a PBYL product with viscosity and WHC comparable to a conventional dairy yogurt [33]. Authors reported that, as expected, the higher was the inclusion of quinoa flour in water, the higher was the viscosity at the end of the process, thus selecting the 35% (*w*/*v*) as the condition corresponding to the optimal texture in the final product.

### 3.2. Synthesis of Exopolisaccharides

Like for the dairy yogurt, the synthesis of EPS during LAB fermentation has been identified as crucial for obtaining optimal texture and sensory characteristics of PBYL products. EPS-synthesis is a strain-dependent metabolic characteristic, affected by the composition of the matrix and fermentation settings [75]. Indeed, the synthesis of EPS is correlated to LAB sugar metabolism, linking the anabolic pathway of EPS production and the catabolic pathway of glycolysis [76].

Different types of EPS are produced by LAB, classified based on their chemical composition. Heteropolysaccharides are formed through linking of different monosaccharides (mainly glucose, rhamnose, or galactose), while homopolysaccharides have only one kind of polymeric unit (mainly glucose or fructose). Heteropolysaccharides are branched, and they have a typical molecular mass between 10^4^–10^6^ Da [77]. Among the heteropolysaccharides, major representative producing strains belong to *Lb. delbrueckii* subsp. *bulgaricus*, *Lb. acidophilus*, *Lactobacillus helveticus*, and *Str. thermophilus*, including the microbial starters used in conventional yogurt production. Homopolysaccharides are formed by extracellular enzymes and are characterized by molecular mass up to 10^6^ Da [12]. Among these, dextrans are polymers of glucose while levans of fructose. Moreover, they are manly produced by strains belonging to *Lactobacillus* (recently reclassified into *Lactiplantibacillus*, *Levilactobacillus*, *Lacticaseibacillus*, *Limosilactibacillus*) [22], *Leuconostoc*, *Streptococcus*, *Weissella*, and *Oenococcus* genera. EPS-producing LAB were previously reported for their relevant contribution to texture improvement and stability of fermented plant-based beverages [2,12,77].

The advantages of EPS enrichment of PBYL are not limited to rheological improvements of the products, but they also include the enhancement of sensory and mouth-feel properties, freeze–thaw stability, and water-holding properties [12].

The EPS composition and amount are the major variables affecting textural properties of fermented products [78]. To standardize EPS final amount and molecular composition, hardly controlled during fermentation processes, EPS supplementation was investigated in dairy yogurt [79]. However, the authors concluded that in-situ production resulted the best approach, due to polymer -proteins interaction during fermentation. Moreover, the use of EPS-producing LAB does not require the mention of additives in the ingredients list so the final product is clean-label [80]. The use of the EPS-producing strain *Weissella confusa* DSM 20194 as starter for the fermentation of a quinoa YL led to the increase of WHC (from 63% ± 3% to 78% ± 3%) and viscosity (from 0.06 Pa·s ± 0.01 Pa·s to 0.49 Pa·s ± 0.09 Pa·s), while slight decreases of viscosity were observed when no EPS-producing LAB strains were inoculated [33]. EPS (dextran), which formation was achieved by the addition of 10% (*w*/*v*) of sucrose, also contributed to stabilize the texture of the PBYL during refrigerated storage. In-situ EPS production corresponding to 40g/L was reported in a quinoa YL fermented by *Weissella cibaria* MG1 [35].

## 4. Nutritional and Functional Aspects: Matrix- and Fermentation-Related Effects

### 4.1. PBYL Variability and Main Differences with Conventional Yogurt

Dairy yogurt provides significant levels of nutrients which depends on milk composition and technologies used during the production process and supplementation with additional ingredients. For example, Greek yogurt, due to the separation of the whey, provides a protein content 2–3 folds higher than the conventional one, this latter containing about 3.4–3.8% of protein. Overall, the main yogurt constituents are proteins with high biological value, lactose, fat, and minerals [81]. Considering that raw materials included in PBYL formulations differ significantly from milk constituents, their nutritional composition is extremely variable.

The use of cereal ingredients, for example, corresponds to PBYL characterized by carbohydrates content which is usually higher than dairy counterparts. Nevertheless, high concentration of fibers can be obtained by using whole grains, as in the case of YL products obtained with oat flakes and brown rice [27,34]. Besides dietary fibers, the use of pseudocereals and legumes also allows to obtain high level of high biological-value proteins, as reported when quinoa, soy, lupin, lentil and chickpea flours were used as the main YL ingredients [11,14,33,41]. Such products were characterized by protein content ranging from 3% to 5% depending on the inclusion rate of the protein source.

Plant proteins are often described as nutritionally incomplete and characterized by low digestibility and bioavailability due to the relatively low amount of essential amino acids and high content of dietary fiber and ANF, respectively [82]. However, amino-acid complementation (mixing cereals and pulses in the same meal), consumption of higher amounts of plant-based products on a more frequent basis [82], and proper bioprocessing of the PB ingredients [15] have been proposed as strategies to maximize the essential amino-acid contents of PB foods.

Moreover, considering that moderate or intense thermal treatments are often required to obtain optimal textural properties (Table 1), their effect on the PBYL protein digestibility should be further investigated.

Phenolic compounds also can be present in non-dairy yogurt, depending on the type of plant-derived ingredients used. For example, high total phenols content was found in quinoa YL products (from 4 to 10 mmol/kg) [33], while relatively lower concentrations were observed when brown rice, oat flakes, and cereal–legume blends were used [13,14,27,34].

Overall, sugars and fat content appear to be very low in the experimental PBYL described in literature, although additional ingredients are often supplemented at industrial level to increase the pleasantness of the commercial formulations, also using oils, fruits purees, syrups, and sweeteners, thus increasing the carbohydrates load of the products.

The large variability in the nutritional composition of PBYL has been observed in products already on the market. Six commercial PBYL products (made from soy, coconut, cashew, almond, and hemp) were analyzed, showing that none of the PBYL had a protein content comparable to dairy benchmark, with the highest value found in soy-based products (up to 4.6 g/100 g) [41]. Fat content was higher in some PBYL due to the use of coconut, cashew, and almond as ingredients. The highest carbohydrates concentration was observed for coconut PBYL product (8.0 g/100 g), the only one containing a higher amount than conventional yogurt (6.1 g/100 g) [41]. The research included two soy PBYL, largely employed as main ingredient of PBYL formulations. Soy has high protein content and optimal amino acids balance, and several compounds with demonstrated functional properties [83]. Indeed, soy PBYL are one of the most suitable alternatives for cholesterol intake reduction in the diet, which could be substituted by phytosterols. The intake of 1–3 g/day of vegetable sterols can reduce serum LDL-cholesterol [84] which is nowadays between 100–400 mg/day in the western diet [85].

### 4.2. Starters and Effect of Fermentation on the Nutritional and Functional PBYL Features

Fermentation is the key biotechnological process to make yogurt. In milk, the acidification caused by LAB fermentation of lactose leads to the synthesis of 80–100 mmol/L of lactic acid through the homofermentative pathway [86]. Furthermore, the enzymatic activities of LAB lead to proteolysis thus increasing the protein digestibility [87] and the concentration of potentially bioactive peptides [88,89]. LAB metabolism also contributes to the peculiar aroma of conventional yogurt through the synthesis of acetaldehyde (circa 0.2 mmol/L). Finally, fermentation affects micronutrient composition and the increase of folic acid was also reported [87]. Yogurt fermentation is conducted by *Str. thermophilus* and *Lb. delbrueckii* subsp. *bulgaricus* representing a perfect example of microbial mutualism in which the first one provides anaerobiosis and other growth-stimulating factors while the latter peptides and free amino acids as nitrogen sources [90].

Several investigations on the application of *Str. thermophilus* and *Lb. delbrueckii* subsp. *bulgaricus* in PBYL production were carried out. Fermentation of soymilk with the above species was conducted with supplementation of 1% of lactose to ensure an optimal bacterial growth and to enhance acid production. LAB fermentation increased the anti-hypertensive activity of the fermented soy matrix, as consequence of the release of peptides with ACE- (Angiotensin-I Converting Enzyme) inhibitory activity through proteolysis of the native soy proteins [28].

However, to find suitable starters for PBYL, the selection of LAB strains different than *Lb. delbrueckii* ssp. *bulgaricus* and *Str. thermophilus* species, more adapted to the vegetable matrices was studied. The use of milk alternatives leads to changes in fermentation process, due to different protein and carbohydrates composition (e.g., absence of lactose), availability of micronutrients, and potential presence of inhibitors (e.g., antimicrobial proteinaceous compounds, polyphenols).

Starters selected for PBYL production should provide a fast acidification that can also prevent contamination from spoilage microorganisms [91], carry out an adequate proteolysis, essential for both nutritional and sensory quality, confer a pleasant aroma [92], possibly improve the texture through EPS-synthesis, and survive at high cell density in refrigerated storage conditions. While the selection criteria for the technological requirements appear simple to be met, since dependent on the acidification and growth performances of the strain under specific environmental and matrix conditions, the criteria related to the potential nutritional and functional effects, such as the release of functional compounds or the capability to affect nutrient bioaccessibility and bioavailability, are currently subjected to a thorough review by the scientific community.

LAB fermentation contributes to the increase of the concentrations of free amino acids and peptides, soluble fibers, and total phenols thus corresponding to higher protein digestibility and increased nutritional value of final PB products [15]. Biological acidification is also associated to the decrease of starch hydrolysis index, mainly due to resistant starch formation, thus decreasing the glycemic index of the final product [93]. Additional advantages of LAB fermentation are represented by the potential decrease of ANF level through specific enzymatic activities (see Section 4.3).

Several studies reported advantages related to the use of LAB strains isolated by the matrix then used for PBYL making, compared to strains of different origin. Lorusso and colleagues compared the performance of *Lactiplantibacillus plantarum* (formerly *Lactobacillus plantarum* [22]) T6B10, previously isolated from spontaneously fermented quinoa, to that of the EPS-producing strain *W. confusa* DSM 20194, used singly as starter for making a quinoa-based YL product [33]. Compared to the allochthonous strain, *La. plantarum* T6B10 allowed the production of a PBYL characterized by higher concentration of lactic acid (84.37 mmol/kg) and total phenols (8.4 mmol/kg). The increase of total phenols concentration is associated with improvement of the antioxidant activity of the matrix. This phenomenon was often reported as generic effect of LAB-induced acidification, that increase phenols solubilization and extractability, but it mainly depends from specific LAB enzymatic activities (e.g., feruroyl esterases), that favour the release of antioxidant phenols from glycosylated and more complex forms, showing a lower activity [94]. The quinoa PBYL product was moreover characterized by high in vitro protein digestibility (IVPD, 84%) and low glycemic index (predicted glycemic index, pGI: 69).

The use of starters previously isolated from the same matrix to be fermented was reported as a successful strategy also for emmer-based YL [32]. In this case, the selection of a proper starter was carried out by comparing the technological properties of LAB strains previously isolated from emmer flour (belonging to *La. plantarum W. confusa*, and *Le. brevis* species) and allochthonous EPS-producing LAB (strains belonging to *W. cibaria*, *La. plantarum*, and *P. pentosaceus* species, previously isolated from wheat sourdoughs). The autochthonous *La. plantarum* 6E resulted the best performing strain allowing the production of an emmer YL product with a low glycemic index (pGI of 70), characterized by relevant concentration of the vitamins thiamin (B1) and niacin (PP). Another *La. plantarum* strain (LP09) was selected among 13 commercial LAB, as starter to produce an oat flake YL product [34], characterized by low starch hydrolysis index (HI = 45, corresponding to a pGI of 64), as the combined results of biological acidification and the high concentration of β-glucan (53%) in the matrix.

*La. plantarum* was often reported as suitable starter because of its robustness under conditions of low pH [95], conferring a competitive advantage against other microorganisms present In the PB matrix. Moreover, it is often associated to the development of ‘dairy’-related flavors (e.g., diacetyl, acetoin, acetaldehyde) [96,97].

A PBYL formulation including a mixture of lentil and chickpea (5% *w*/*v* each) and rice flours (10% *w*/*v*) was proposed to overcome the poor protein quality usually found in commercial PB alternatives to milk products [14]. The formulation also contained a high level of dietary fibers (4% *w*/*v*). The gelatinized flour suspension was fermented by a mixed culture including *La. plantarum* DSM33326 and *Le. brevis* DSM33325. Fermentation caused an increase of the total free amino acids concentration (65% higher than the unfermented matrix). As consequence of the intense proteolysis, the IVPD of the fermented product was 79.5%. Additionally, LAB proteolysis further improved the nutritional indexes values, thanks to the hydrolysis of protein sequences resistant to the activity of the digestive enzymes. Overall, significant increases of protein chemical score (CS), essential amino acids index (EAAI), protein efficiency ratio (PER), biological value (BV), and nutritional index (NI) were found after fermentation. Also in this case, YL product was moreover characterized by a low glycemic index (pGI of 53). Fermentation caused also a significant increase of GABA (found at final concentration of circa 110 mg/100 mL), and of the antioxidant activity [14].

GABA is involved in several human physiologic conditions, such as neurotransmission, and it is a hypertension modulator [98]. Several studies reported reduction of hypertension, prevention of cancer cell proliferation, and modulation of blood cholesterol levels following regular administration of GABA in humans and animals [99]. Although legumes can have already high level of GABA [98,99,100], its content can increase due to LAB with glutamate-decarboxylase activity. This feature was already proposed for the in situ enrichment of fermented milks [101].

A lower amount of digestible proteins in soy-based yogurt compared to soymilk was also observed [36]. This outcome, however, was probably due to the duration of fermentation, less than 6 h, a time markedly lower than that used in all the above-mentioned studies, from 16 to 24 h. Indeed, it was already reported that proteases activity is poor during the early fermentation stage, in which LAB preferentially use the available free amino acids [102].

### 4.3. Degradation of Anti-Nutritional Compounds

The nutritional value of PBYL could be affected by the presence of several ANF (e.g., raffinose, phytic acid, condensed tannins, alkaloids, lectins, pyrimidine glycosides, and protease inhibitors), often present at high concentration in plant matrices, such as legumes and pseudocereals or whole grains.

As mentioned before, fermentation by LAB is effective in decreasing the amount of specific ANF in these substrates [15,103]. Generally, the decrease of α-galactosides (e.g., raffinose), phytic acid, condensed tannins, and trypsin inhibitors are observed during sourdough fermentation as the result of the endogenous enzymatic activity of plants and activity of LAB [15,104].

During fermentation, phytase and other phosphatase—activated by the pH decrease or belonging to LAB—could degrade phytic acid, which renders minerals and inorganic phosphate unavailable through chelation [105,106]. Trypsin inhibitors are commonly considered an ANF due to a correlation with low protein digestibility, although inactivated by thermal treatments, if included in food preparation. Saponins and condensed tannins are also correlated to protein digestibility decrease but their role in nutrition is under debate due to some reported beneficial activity. The route to degrade tannins by LAB implies that tannic acid is hydrolyzed to gallic acid and glucose, and the gallic acid formed is further decarboxylated to pyrogallol [94]. This pathway implies the activity of tannase and gallate decarboxylases, whose presence was previously documented, for example, in *La. plantarum* [107]. Legumes contain relevant concentrations of α-galactosides which are not degraded in the upper gastrointestinal tract and fermented in the large intestine, causing gastrointestinal symptoms, including abdominal discomfort, flatulence, and diarrhea [108]. α-galactosidase activity of LAB was effective in reducing their content during fermentation of different legume flours [109,110,111]. Relevant decreases of ANF (condensed tannins, raffinose, phytic acid, and saponins) during PBYL fermentation (cereals-legumes blend) with selected LAB were documented in recent studies [14].

### 4.4. Use of Sprouted Grains

The use of sprouted grains and derived flours as PBYL ingredients was recently investigated. The germination process, largely employed in malt production, is associated with the increase of the enzymatic activities of the seed embryo [112] causing the release of fermentable sugars, peptides and amino acids from polymeric molecules and with the degradation of ANF factors [113,114], besides improving the sensory properties of several plant matrices.

Fermentation of sprouted soy by *Le. brevis* KCTC 3320 influenced the concentration of GABA. The glutamate decarboxylase activity and GABA concentration were monitored during 72 h of fermentation of sprouted soy. After 60 h, the highest concentration of GABA (120.38 mg/100 mL) was detected, together with a significant increase of isoflavone aglycones (daidzein, glycitein, and genistein), resulting from the high β-glucosidase activity, and increase of the antioxidant activity. This process resulted in a functional PYBL [37].

The length of the germination process (soaking and incubation for 48 or 96 h) affected the concentration of bioactive compounds and biological activity of a brown rice YL product [27]. A synergic effect of germination and fermentation on GABA synthesis was observed (final concentration 1.86 mg/100 g). Moreover, the combination of both processes increased antioxidant and ACE)-inhibitory activities, respectively correlated to the phenolic compounds and peptides released during germination and LAB fermentation.

### 4.5. PBYL as Probiotic Carriers

Yogurt is considered the optimal means for delivering probiotic microorganisms in the diet. Despite probiotics definition is currently under debate, FAO and WHO identify probiotics as live microorganisms which can pass alive the digestive tract and colonize the bowel and can provide health benefits for the host when consumed in appropriate amounts [115].

Two different approaches for probiotics in PBYL are currently considered in the literature: (1) microorganisms with proved probiotic features can be selected also for the technological properties and used as starter for YL fermentation; (2) probiotics can be added to the YL, after or before it undergoes fermentation with starters chosen for the proper technological characteristics. In both cases the adaptability of the probiotic to the PB environment is essential [21,116]. The suitability of PBYL as potential carrier of probiotics was reported in cereal- and legume- based YL products [14,32,50,117]. A probiotic strain of *Lacticaseibacillus rhamnosus*, inoculated together with other LAB strains selected as starters for fermentation, was able to survive at cell density higher than 8 log10 cfu/mL in emmer and cereal/legume YL during 30 days of cold storage [14,32].

The probiotic potential of a peanut YL product fermented by seven probiotic LAB strains (*Le. brevis* MTCC1750, *Le. brevis* MTCC1423, *Limosilactobacillus fermentum* MTCC903, *Li. fermentum* MTCC1745, *La. plantarum* MTCC6160, *La. plantarum* MTCC1407, and *Enterococcus faecalis* T110) was investigated [38]. *En. faecalis* T110 was selected based on preliminary sensory analysis due to the less acidic flavor. Inoculum entity and fermentation time were the most important factors ensuring the required number of viable cells in the final product. Besides inoculum and fermentation time, optimal fermentation temperature was also defined at 37 °C, since allowed to obtain higher viable cell density compared to 43 °C, confirming the previous results of Shortt [118].

## 5. Sensory Profile and Consumer Acceptance

PBYL sensory properties are strongly affected by their formulations. Plant matrices are often characterized by typical bitter, beany, astringent, herbaceous taste, and an odor perceived as unusual by regular consumers of dairy yogurt.

The sensory properties of soy-derived YL products, the more consolidated and widespread PB alternative to dairy yogurt, have been investigated in depth. Typically, these soy YL are fermented with strains of *Str. thermophilus* and *Lacticaseibacillus casei* to resemble conventional yogurt. Nevertheless, compared to conventional yogurt, moderate perceptible “beany” and “raisin” aromas, such as “bitter taste” and “astringency” were found in soy formulations [29]. The beany flavor of soy is one of the main challenges in formulating soy or legume-based YL products, for which the addition of flavoring agents might not always be sufficient [119]. The inclusion of strawberry or orange jam in relevant amount (30%) in a soy YL product, improved their overall acceptability, flavor, aroma, and taste compared to the unflavored control. Nevertheless, depending on the cultural background, some consumers were able to distinguish the sensory properties among samples while others did not detect differences, probably due to the familiarity of specific groups with “beany“ flavor [26]. Fruit syrup supplementation led to a lower perception of “sourness“ and “beany” soy attributes in a strawberry soy YL product fermented by *Bifidobacterium longum* SPM1205, *La. plantarum* CBT1209, and *P. pentosaceus* CBT SL4 [39]. The positive influence of flavoring in PBYL was confirmed in a recent study conducted in Ireland, in which flavored PBYL products (soy-and coconut-based) were found similar to dairy yogurt in all sensory descriptors results (appearance, odor, flavor, texture, and overall acceptability) [41]. Sugars and fruity aromas probably played the main role of masking unusual smells and flavors of the products.

Cereal based YL seems also to possess specific sensory attributes depending on the species considered. Unpleasant odors and flavors have been associated to the use of raw, not processed or not refined forms. The ‘bitterness’ and ‘astringency’, for example, could derive from phenolic compounds found in the outer layers of whole grains [120]. Oat, although largely used as main ingredient for making commercial PBYL (Table 1), rapidly develops green and bitter taste if a suitable heating treatment, aimed at lipolytic enzymes inactivation, does not follow the harvesting [121].

Emmer YL products were characterized by ‘earthy’, ‘dairy’, ‘cereal’, ‘savory’ or ‘beany’ attributes. Quinoa YL were assessed using similar descriptors and all PBYL tested were characterized by the presence of particles and a high ‘astringency’ perception [33].

Overall, fermentation was reported as able to decrease unpleasant flavor of different flours such as ‘bitter taste’, ‘beany’, and ‘astringency’ [14,32,33].

Considering that sensory perceptions change during mastication, the temporal dominance of sensations (TDS) analysis represents an effective method to describe sensory profiles for novel products like PBYL. TDS shows the sequence of the dominant sensations during a defined period of sensory analysis, focusing on the most important perceptions [122]. TDS was used to evaluate five commercial oat YL products [42]. Overall, dairy products were considered equilibrate without a strongly dominant attribute. Nevertheless, the most important perceptions until the end of mastication were found to be ‘creamy’ and ‘thinness’/’wateriness’ in yogurt and PBYL, respectively. Moreover, ‘creaminess’ dominance was found also for two out of five PBYL. This perception, which was previously correlated to fat content in dairy product [123], was attributed to pectin inclusion in oat YL formulation [42].

Sensory perceptions are related to the biochemical features of the food matrix, and volatile compounds, in particular, are responsible for the odor perception.

The volatile organic compounds profile of PBYL products could be characterized by the same compounds of dairy counterparts. Among these, diacetyl, acetoin, acetaldehyde, acetone, and ethanol are particularly important. Both acetoin and diacetyl confer buttery odors, caramel and sweet flavors while alcohol is recognized as fruity and floral ethyl esters precursor, when reacting with fatty acids [124]. Moreover, acetaldehyde concentration, one of the most important volatiles of conventional yogurt [125], could largely vary in PBYL.

## 6. Shelf-Life

PBYL are intended to be consumed after a storage period in refrigerated conditions, similarly to the dairy counterpart. In these conditions, the persistence of optimal textural and sensory characteristics for a relatively long period is considered crucial. Some of the changes occurring during storage include aqueous phase separation, loss of viscosity, appearance of off odors, and overall intensification of acidic smell and flavor.

The pH of fermented PBYL is usually lower than 4.5 [7] and, similarly to conventional yogurt, post-acidification due to the viable LAB activity is typically observed during refrigerated storage [14,28,32,33,34,126]. Overall, the pH decrease ranged from 0.5 to 1.0 pH units. As expected, a moderate decrease in LAB viability was also observed during storage, although densities higher than 8 log10 cfu/mL were also found after 20–30 days at 4 °C [14,32,33].

The viability of LAB during different storage conditions was also tested in a millet -based probiotic YL. Two different combinations of time/temperature were compared: refrigeration at 4 °C for 8 weeks and at room temperature (22 °C) for 5 days. Although the viability of *Str. thermophilus* drastically decreased after 20 days in refrigerated conditions and as fast as 2 days at room temperature, the probiotic *Lac. rhamnosus* GR-1 still showed cell densities higher than 8 log10 cfu/mL at the end of the storage period in both the conditions [30].

To increase the viability during storage conditions, one possible strategy could be to supplement prebiotics.

Donkor and colleagues monitored different parameters, including pH, organic acids, viability of probiotics, and sensory acceptability in soy YL fermented by *Str. thermophilus* St1342, *L. delbrueckii* ssp. *bulgaricus* Lb1466 with or without the addition of the probiotic strains *Lb. acidophilus* L10, *Lac. casei* L26, and *Bifidobacterium animalis* B94 [126]. Inulin (2%) or raffinose and glucose (1% each) were added to investigate the effect on probiotic viability during storage. As previously observed in conventional yogurt, the supplementation of inulin or sugars stimulated a slight growth of the probiotic bacteria during storage [127].

Strong acidic conditions, presence of bacteriocins and other antimicrobial compounds synthesized by LAB, as well as the high cell density of the LAB starters are all factors playing against PBYL contamination by spoilage microorganisms [14].

Considering the high water activity and the overall susceptibility of plant ingredients to spoilage, besides microbial contamination, other physical-chemical modification of the PBYL products from production to consumption time, can occur. Color modification during storage of commercial soy PBYL were prevented when red radish extract was added while red radish and hibiscus microencapsulated extracts were considered the most stable during storage [40]. Moreover, betalains and anthocyanins released in the PBYL during storage had a protective action against oxidation.

## 7. Final Remarks

PBYL represent the most versatile group of functional foods already widespread through the global market. The possibility to employ and mix different plant ingredients to modulate the nutritional composition (proteins, sugars, fat, dietary fibers concentration) enable the creation of PBYL formulation to meet specific needs of the modern consumer, from the nutritional, sensory or ethical points of view. Fermentation has been recognized as fundamental to achieve a proper sensory profile, but it also represents an effective biotechnological tool to improve nutritional and functional features of the plant-based ingredients. PBYL are intended as carrier of LAB and probiotics at high cell density, a functional feature that can be further accompanied by the presence of functional compounds, synthesized in situ by the starters or directly provided by the PB ingredients. Being similar to the milk yogurt, traditionally associated with well-known health properties, their acceptance as functional foods is greatly facilitated. Several efforts have been made by academic and food industry research to overcome the issues related to the structure and the sensory profiles of PBYL, and further updates are shortly expected.

## Figures and Tables

**Table 1:** Main ingredients, bioprocessing options, and microbial starters employed in plant-based yogurt like production.

Main Ingredients	Starters	Texture Processing, Structuring Agents	Development Level	Reference
Oat protein concentrate (15% *w*/*w*)	*Streptococcus thermophilus* and *Lactobacillus delbrueckii* subsp. *bulgaricus* (commercial strains for yogurt production)	Heat treatment at 90 °C for 30 min	Experimental	[23]
Potato protein isolate (5% *w*/*v*)	*Streptococcus thermophilus* and *Lactobacillus delbrueckii* subsp. *bulgaricus* (commercial strains for yogurt production)	High-pressure homogenization (200 MPa)	Experimental	[24]
Pea protein isolate (10% *w*/*w*)	*Streptococcus thermophilus* and *Lactobacillus delbrueckii* subsp. *bulgaricus* (commercial strains for yogurt production)	Heat treatment 60°C for 60min and high-pressure homogenisation (3 MPa)	Experimental	[25]
Soymilk (6.8% solids)	*Streptococcus thermophilus* and *Lactobacillus delbrueckii* subsp. *bulgaricus* (commercial strains for yogurt production)	Concentration (heat treatment at 90 °C for 15 min), addition of strawberry or orange jam (30% *w*/*w*)	Experimental	[26]
Brown rice, soaked rice, or germinated rice (22% *w*/*v*)	Commercial thermophilic starters	Gelatin supplementation, heat treatment at 95°C for 30min, filtration	Experimental	[27]
Soymilk	*Streptococcus thermophilus* St1342, *Lactobacillus delbrueckii* subsp. *bulgaricus* Lb1466 and a probiotic strain *(Lactobacillus acidophilus* L10, *Lacticaseibacillus paracasei* L26, *Bifidobacterium lactis* B94)	Heat treatment at 90 °C for 30 min	Experimental	[28]
Defatted soy flour (11.6% *w*/*w*)	*Streptococcus thermophilus* ATCC 19987 and *Lacticaseibacillus casei* ATCC 393	Heat treatment at 121 °C for 15 min and supplementation with gelatin	Experimental	[29]
Millet flour (8% *w*/*v*)	*Lacticaseibacillus rhamnosus* GR-1 and *Streptococcus thermophilus* C106	Heat treatment at 90-95 °C for 60 min	Experimental	[30]
Almond (8% *w*/*w*)	*Limosilactobacillus reuteri* ATCC 55730 (probiotic) and *Streptococcus thermophilus* CECT 986	High pressure homogenisation (172 MPa for 2-4 sec) and heat treatment at 85 °C for 30 min	Experimental	[31]
Emmer flour (30% *w*/*v*)	*Lactiplantibacillus plantarum* 6E, *Lacticaseibacillus rhamnosus* SP1, *Weissella cibaria* WC4 (EPS-producer)	Starch gelatinization at 60° for 30 min, use of EPS-producer LAB strain	Experimental	[32]
Quinoa (35% *w*/*v*)	*Lactiplantibacillus plantarum* T6B10, *Lacticaseibacillus rhamnosus* SP1 (probiotic), *Weissella confusa* DSM 20194, (EPS-producer)	Starch gelatinization at 63 °C for ca. 19 min.	Experimental	[33]
Lupin protein isolate (2% *w*/*v*)	*Lactiplantibacillus plantarum* TMW 1.460 and TMW 1.1468, or *Pediococcus pentosaceus* BGT B34 and *Levilactobacillus brevis* BGT L150	Heat treatment (140 °C for 10 s or 80 °C for 60 s) and EPS-producer LAB strain	Experimental	[11]
Oat flakes (25% *w*/*w*)	*Lactiplantibacillus plantarum* LP09	Enzymatic treatments (xylanase and α-amylase)	Experimental	[34]
Rice (10% *w*/*w*), lentil (5% *w*/*w*), and chickpea (5% *w*/*w*) flours	*Lactiplantibacillus plantarum* DSM33326, *Levilactobacillus brevis* DSM33325, *Lacticaseibacillus rhamnosus* SP1 (probiotic)	Heat treatment at 80°C for 15 min	Experimental	[14]
Quinoa flour (14.3% *w*/*w*)	*Weissella cibaria* MG1 (EPS producer)	Heat treatment at 121 °C for 15 min, α-amylase and protease treatments, high-pressure homogenisation (180 MPa)	Experimental	[35]
Soy (10% *w*/*v*)	*Lactiplantibacillus plantarum* B1-6	Heat treatment at 108 °C for 15 min	Experimental	[36]
Soy, soaked soy, or germinated soy (10% *w*/*v*)	*Levilactobacillus brevis* KCTC 3320	Heat treatment at 121 °C for 15 min	Experimental	[37]
Peanut (16.7% *w*/*w*)	*Enterococcus faecalis* T110 (probiotic)	Heat treatment in autoclave at 121 °C and 15 psi for 3–5 min	Experimental	[38]
Soymilk (12.5% *w*/*w*)	*Bifidobacterium longum* SPM1205	Heat treatment at 95 °C for 5 min, supplementation with agar, strawberry syrup (20% *w*/*w*) and 0.05% (*w*/*w*) of freeze-dried diced strawberry	Experimental	[39]
Soy and a pigment rich extract (red beetroot, hibiscus, opuntia, red radish)	*-*	-	Experimental	[40]
Hulled soy beans (7.9% *w*/*v*)	*Streptococcus thermophilus* and *Lactobacillus delbrueckii* subsp. *bulgaricus* (commercial strains for yogurt production)	Supplementation with pectin	Commercial	[41]
Hulled soy beans (9% *w*/*v*)	*Streptococcus thermophilus* and *Lactobacillus delbrueckii* subsp. *bulgaricus* (commercial strains for yogurt production)	-	Commercial	[41]
Coconut cream (20% *w*/*v*) and modified maize starch	*Streptococcus thermophilus* and *Lactobacillus delbrueckii* subsp. *bulgaricus* (commercial strains for yogurt production)	Supplementation with pectin	Commercial	[41]
Cashew “milk” (97% *v*/*v*) and tapioca starch	*Streptococcus thermophilus* and *Lactobacillus delbrueckii* subsp. *bulgaricus* (commercial strains for yogurt production)	Supplementation with carob gum	Commercial	[41]
Almond “milk” (95% *v*/*v*) and tapioca starch	*Streptococcus thermophilus* and *Lactobacillus delbrueckii* subsp. *bulgaricus* (commercial strains for yogurt production)	Supplementation with carob gum	Commercial	[41]
Hemp juice 96% (water, hemp seed 3% *w*/*v*) and rice starch	Selected strain of *Bifidobacterium* and *Lactobacillus acidophilus*	Supplementation with agar	Commercial	[41]
Oat 12% (*w*/*v*)	-	Supplementation with potato starch and potato protein	Commercial	[42]
Oat 8.5% (*w*/*v*)	-	Supplementation with modified starch, pectin	Commercial	[42]
Oat 8% (*w*/*v*)	-	Supplementation with potato protein, starch (corn, potato), pectin	Commercial	[42]
Oat 12% (*w*/*v*)	-	Supplementation with potato protein, tapioca starch, potato starch, xanthan, locust bean gum	Commercial	[42]
Oat	-	Supplementation with pea protein, modified potato starch	Commercial	[42]
Oat 12% (w/v) (OATLY^®^)	Commercial strains for yogurt production	Supplementation with potato starch	Commercial (Oatly AB, Sweden)	[43]
Soy 10.7% (*w*/*v*) (ALPRO ^®^)	*Streptococcus thermophilus* and *Lactobacillus delbrueckii* subsp. *bulgaricus* (commercial strains for yogurt production)	Supplementation with pectin	Commercial (Alpro, Belgium)	[44]
Oat 8% (*w*/*v*) (YOSA ^®^)	*Bifidobacterium* BB12 and *Lacticaseibacillus rhamnosus* GG	Supplementation with pectin	Commercial (Fazer Oy, Finland)	[45]

## Data Availability

Not applicable.

## Abbreviations

ACE: angiotensin-I converting enzyme; ANF: antinutritional factors; BV: biological value; CFU: colony forming units; CS: protein chemical score; EAAI: essential amino acids index; EPS: exopolysaccharides; GABA: γ-aminoburyric acid; IVPD: in vitro protein digestibility; LAB: lactic acid bacteria; NI: Nutritional Index; OPC: oat protein concentrate; OPI: oat protein isolate; PB: plant-based; PBYL: plant-based yogurt-like; PER: protein efficiency ratio; pGI: predicted glycemic index; QPM: Quality Protein Maize; TDS: temporal dominance of sensations; UHT: ultra-high temperature; UHPH: ultra-high pressure homogenization; VOC: volatile organic compounds; WHC: water holding capacity; YL: yogurt-like.
